# CRISPR–Cas Systems Associated with Electrolyte-Gated Graphene-Based Transistors: How They Work and How to Combine Them

**DOI:** 10.3390/bios14110541

**Published:** 2024-11-07

**Authors:** Pierre Guermonprez, Pierre Nioche, Louis Renaud, Nicolas Battaglini, Sébastien Sanaur, Eric Krejci, Benoît Piro

**Affiliations:** 1 ITODYS, CNRS, Université Paris Cité, F-75006 Paris, France; pierre.guermonprez@u-paris.fr (P.G.); nicolas.battaglini@u-paris.fr (N.B.); 2 INSERM US 36|CNRS UAR 2009, Structural and Molecular Analysis Platform, Université Paris Cité, F-75006 Paris, France; pierre.nioche@parisdescartes.fr; 3 INSERM U1124, Université Paris Cité, F-75006 Paris, France; 4 Institut des Nanotechnologies de Lyon INL-UMR5270, Université Lyon 1, F-69622 Villeurbanne, France; louis.renaud@univ-lyon1.fr; 5 Department of Flexible Electronics, Institut Mines-Telecom, Mines Saint-Étienne, F-13541 Gardanne, France; sanaur@emse.fr; 6 CNRS, ENS Paris Saclay, Centre Borelli UMR 9010, Université Paris Cité, F-75006 Paris, France; eric.krejci@parisdescartes.fr

**Keywords:** CRISPR/Cas13a, electrolyte-gated transistor, graphene, DNA, RNA, transduction, sensor

## Abstract

In this review, recent advances in the combination of CRISPR–Cas systems with graphene-based electrolyte-gated transistors are discussed in detail. In the first part, the functioning of CRISPR–Cas systems is briefly explained, as well as the most common ways to convert their molecular activity into measurable signals. Other than optical means, conventional electrochemical transducers are also developed. However, it seems that the incorporation of CRISPR/Cas systems into transistor devices could be extremely powerful, as the former provides molecular amplification, while the latter provides electrical amplification; combined, the two could help to advance in terms of sensitivity and compete with conventional PCR assays. Today, organic transistors suffer from poor stability in biological media, whereas graphene materials perform better by being extremely sensitive to their chemical environment and being stable. The need for fast and inexpensive sensors to detect viral RNA arose on the occasion of the COVID-19 crisis, but many other RNA viruses are of interest, such as dengue, hepatitis C, hepatitis E, West Nile fever, Ebola, and polio, for which detection means are needed.

## 1. Introduction

Infectious diseases occur when an external agent enters the human body, whether in the form of bacteria, fungi, parasites, or viruses. Among the latter include SARS-Cov-2 but also Ebola, Zika, the human immunodeficiency virus (HIV), the various hepatitis viruses, and the influenza virus. Despite constant progress in detecting them in the early stages of infection, established and emerging viruses remain major causes of human pathologies, with dramatic consequences: in addition to acute illnesses, viruses are responsible for at least 15–20% of human cancers (hepatitis viruses, papillomaviruses, etc.), and are implicated in numerous neurological disorders and chronic diseases [1].

One of the many challenges in the fight against virus-mediated diseases is the ability to detect viruses in the early stages of infection. It is therefore necessary to find alternatives to conventional laboratory detection methods, moving towards rapid, accurate, and easy-to-use detection schemes, otherwise known as Point-Of-Care (POC) devices. These POC devices, whose development has increased sharply in recent years, meet certain criteria defined by the WHO (Word Health Organization) under the REASSURED guideline: real-time connectivity, ease of specimen collection, affordable, sensitive, specific, user-friendly, rapid, robust, equipment-free, and deliverable to end users [2].

In recent years, CRISPR–Cas systems have emerged as innovative new tools in molecular diagnostics and have received increasing attention as a powerful tool in the fight against viral infections. These systems are capable of recognizing a specific nucleic acid sequence which activates an enzymatic hydrolysis activity. For example, one CRISPR–Cas13a complex can cleave up to 100 RNA strands. This amplification property has the potential to address the major challenges associated with nucleic acid detection and improve the insufficient sensitivity of current alternatives to qPCR (quantitative Polymerase Chain Reaction). The first Cas13- or Cas12-based detection platforms (Specific High-Sensitivity Enzymatic Report UnlOCKing: SHERLOCK), used for the detection of SARS-CoV-2, demonstrated high specificity and sensitivity compared to conventional techniques [3]. However, this approach has certain limitations, notably, the presence of multi-step nucleic acid amplification and additional fluorescent labeling, which can affect the precise quantification of the RNA target and increase the reaction time and the number of reagents required.

To add another amplification stage to that of the CRISPR–Cas, transistors and, more particularly, Electrolyte-Gated Field Effect Transistors (EGFETs) offer new possibilities due to their ability to amplify a local electrostatic event into a measurable current or voltage change, in an aqueous environment. In recent years, EGFET biosensors, and, in particular, those based on graphene (EGGFETs), have received increasing attention because they benefit from the 2D (two-dimensional) nature of graphene’s very thin, sensitive layer, the high mobility of its charge carriers, and its excellent biocompatibility with biological samples and their stability in biological solutions. As a result, EGGFET biosensors are seen as ideal platforms for enhancing the performance of CRISPR–Cas-mediated devices.

This article reviews the current state of the art in this field. First, the mechanism of a viral infection is explained, along with the various specific biomarkers expressed during such infections and their respective detection techniques. The value of CRISPR–Cas complexes for nucleic acid detection is then presented. Next, the various biosensors, and, in particular, EGFETs, are introduced, along with their characteristics. Their suitability for the detection of biological species in solution is discussed, and several examples of nucleic acid detection are given. Finally, the various biosensors based on EGFETs in combination with CRISPR–Cas systems are presented, reviewed, and discussed.

## 2. Current Detection Methods of Viral Infections

Viruses are pathogenic organisms that affect human health to varying degrees, ranging from simple discomfort and mild symptoms such as fever and headaches to respiratory difficulties that can lead to irreversible trauma [4]. Furthermore, an uncontrolled viral infection can rapidly turn into a pandemic if left unchecked, with health, social, and economic consequences. A rapid and robust means of detection is therefore essential to combat the spread of viruses. The mechanisms of infection by a viral organism, as well as current detection methods against these pathogens, are detailed below.

### 2.1. Current Detection Methods for Viral Infections

#### 2.1.1. General Information on Detecting Viral Infections

During the course of an infectious disease, there are a number of techniques for detecting the presence of an invading virus; these fall into several categories. Direct methods, such as cell culture and microscopy, enable the presence of the virus to be observed visually. Other methods, which can be described as indirect, exploit the presence of various biological compounds associated with a viral infection, known as viral biomarkers. The main biomarkers are antigens and antibodies, detected by immunological methods, as well as genomic RNA and/or DNA, detectable by nucleic acid amplification techniques, with the most widespread being PCR (Polymerase Chain Reaction). These indirect methods appear to be the most suitable for the development of sensitive, specific, portable, rapid, and easy-to-use diagnostic platforms [5].

#### 2.1.2. Nucleic Acid Amplification and Detection Methods

In the event of a viral infection, DNA detection is tantamount to knowing whether the host has been infected by the virus. It does not give precise temporal information on the stage of the disease or its evolution; however, the DNA will always be present, even once the infection is over. Viral RNA, on the other hand, exists as messenger RNA to code viral proteins, thanks to ribosomes. Its lifespan is very short, from a few minutes to a few days at most, due to the RNA enzymes present in the cytoplasm, which degrade it. Thus, the presence of viral RNA indicates an ongoing infection. Quantifying and monitoring RNA concentration can also help track the evolution of a disease. This is why the detection of RNA is widely used to detect and monitor the evolution of viral infections [6].

### 2.2. The Use of CRISPR–Cas Complexes as a New Means of Nucleic Acid Detection

**Figure 1:**
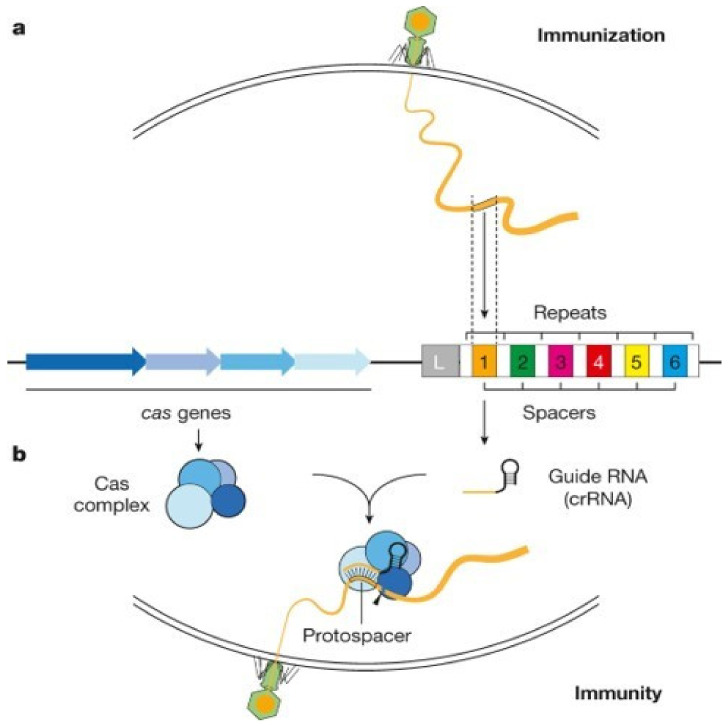
Immune Response of CRISPR–Cas Systems for (**a**) the immunization stage and for (**b**) the immunity stage. Adapted from [10] with permission from Springer Nature.

The discovery of CRISPR dates back to 1987 in Japan when repeated DNA sequences were observed in the genome of the bacterium Escherichia Coli [7]. This CRISPR (Clustered Regularly Interspaced Spacer with Palindromic Repeats) array is composed of short, repeated nucleotide sequences called repeats, interspersed with short, variable, and unique nucleotide sequences called spacers, which originate from the genetic material of viruses or phages involved in previous infections (Figure 1) [8,9]. These spacers are central to the CRISPR defense mechanism, as they provide specific immunity against phages or viruses that possess a complementary DNA sequence. Indeed, adjacent to the CRISPR network are a series of genes encoding Cas proteins (CRISPR-associated). The pooling of the CRISPR network and Cas proteins enables the creation of CRISPR–Cas systems, which provide the bacterium with an immune and adaptive defense.

The immune response of the CRISPR–Cas system can be described in two stages. In the first stage (immunization), viral genome sequences are captured when foreign DNA enters the cell so that the CRISPR network is enriched with a new virus-specific sequence (Figure 1a) [10]. In the second stage (immunity), the CRISPR array is transcribed in short RNAs (Figure 1b) called crRNAs (CRISPR RNAs) or guide RNAs. Then, the crRNAs bind to the Cas proteins and act as an antisense guide to direct these Cas nucleases toward the foreign genetic material. When the DNA sequence complementary to the guide RNA sequence is detected by hybridization of the two nucleic acid chains, the CRISPR–Cas complex triggers its nuclease activity, starting to hydrolyze the invading DNA strand.

The greatest strength of this technology lies in its specificity: the CRISPR–Cas complex can scan an entire DNA genome in just a few minutes and bind very specifically to the complementary sequence of its guide RNA [11]. This technology is now becoming increasingly widespread in the field of diagnostics and is helping to boost sensor performance. Different types of nucleic acid (RNA or DNA) detection based on CRISPR–Cas are presented below.

#### 2.2.1. Classification of CRISPR–Cas Systems and Complexes of Interest

**Figure 2:**
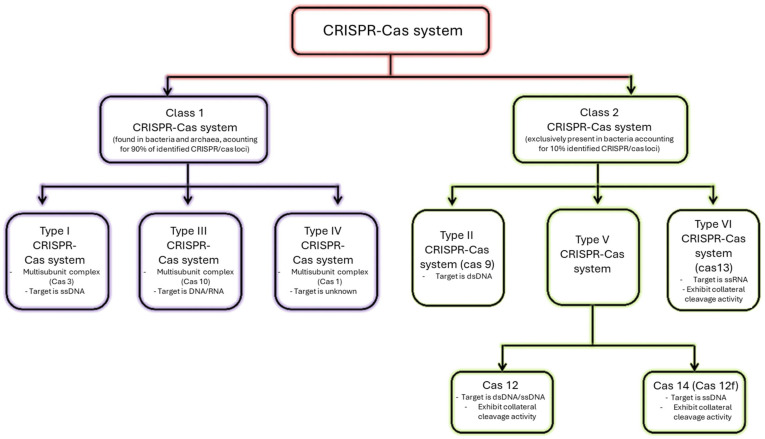
Simplified classification of current CRISPR–Cas systems. Adapted from [14] under CC BY license.

CRISPR–Cas systems are distinguished into two classes and six types (Figure 2) [11,12,13]. Class 1 CRISPR–Cas systems are composed of multiple proteins. These are the most abundant, accounting for 90% of CRISPR–Cas systems [12]. Class 2 CRISPR–Cas systems are made of a single protein and account for 10% of CRISPR–Cas systems.

The CRISPR–Cas tools most widely used and simplest to implement are those of class 2 (type II, type V, and type VI). The first discovery was CRISPR–Cas9, by Jennifer Doudna and Emmanuelle Charpentier in 2012, revolutionizing genome editing [15]. The main difference with the genome editing systems used at the time lies in the recognition of the sequence to be edited, is that CRISPR–Cas relies on RNA and not on proteins [16,17]. Given that it is much easier to produce synthetic RNAs than proteins, CRISPR–Cas9 has quickly become indispensable for genome editing; Jennifer Doudna and Emmanuelle Charpentier won the Nobel Prize in 2020 [18].

The high specificity of CRISPR–Cas9’s guide RNA towards the target DNA has enabled it to be used in the field of biosensors, as Pardee et al. who combined isothermal gene amplification (LAMP) with the nuclease activity of CRISPR–Cas9 to detect the Zika virus [19]. The specificity of CRISPR–Cas technology enabled them to detect and differentiate between American and African strains of the Zika virus, with a resolution of just one nucleotide.

**Figure 3:**
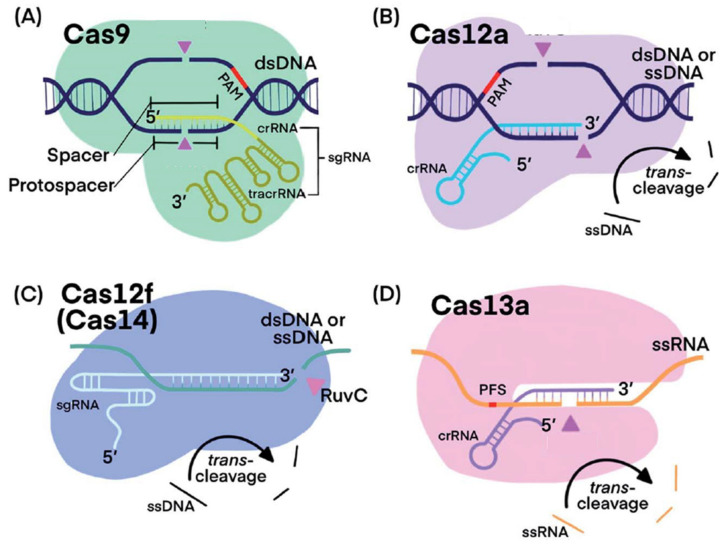
Fundamental functioning principles of (**A**) CRISPR–Cas9, (**B**) Cas12a, (**C**) Cas12f (Cas14), and (**D**) Cas13a systems. Pink triangles indicate *cis*-cleavage sites. PAM are protospacers. Adapted from [24] under Creative Commons Attribution 3.0 Unported License.

After 2014, other class 2 CRISPR–Cas systems came to light, with the discovery of the Cas12, Cas13, and Cas14 enzymes [20,21,22,23]. Unlike Cas9, these enzymes have two distinct catalytic sites that grant them non-specific RNAse (Cas13) or DNAse (Cas12 and Cas14) activity [19]. In other words, once activated, Cas13 can non-selectively cut any RNA strand it encounters, leading to the non-specific degradation of all RNAs in solution (Figure 3). Cas12 and Cas14 have a similar nuclease activity towards DNA strands.

These discoveries have had a major impact on the nucleic acid detection methods used to date. By combining the specificity of CRISPR–Cas systems with isothermal nucleic acid amplification methods such as RPA (recombinase polymerase amplification), it becomes possible to create specific, rapid, sensitive, and portable devices for DNA or RNA detection [25,26,27]. Some of these new platforms, which use Cas13 such as SHERLOCK, CARMEN, or CARVER are detailed below [28,29,30,31].

#### 2.2.2. SHERLOCK: Specific High Sensitivity Reporter Unlocking

The SHERLOCK (Specific High Sensitivity Reporter unLOCKing) method is the first biosensor based on a CRISPR–Cas system to have been implemented. First, the DNA or RNA fragments to be analyzed are amplified by RPA (if the target is RNA, a reverse transcription step is required prior to RPA). Next, these amplified DNA fragments are transcribed back into RNA (as CRISPR–Cas13 only recognizes RNAs) and brought into contact with the CRISPR–Cas13 which has been previously programmed by a crRNA complementary to the target RNA.

**Figure 4:**
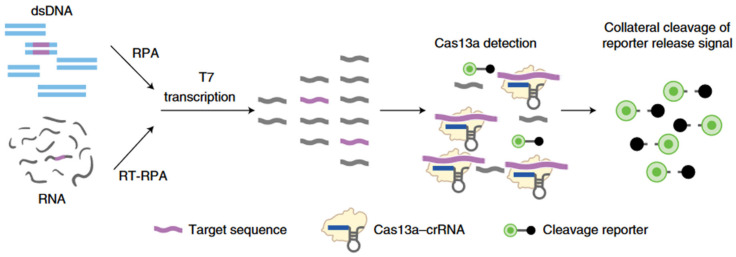
Schematic diagram of the SHERLOCK method. Adapted from [3] under CC BY-NC-SA 4.0 license.

This cleavage is transduced optically as shown in Figure 4, whereupon cleavage, the RNA probe is split into two parts, resulting in the separation of the fluorescence quencher from the fluorophore, so that the fluorophore signal becomes visible.

**Figure 5:**
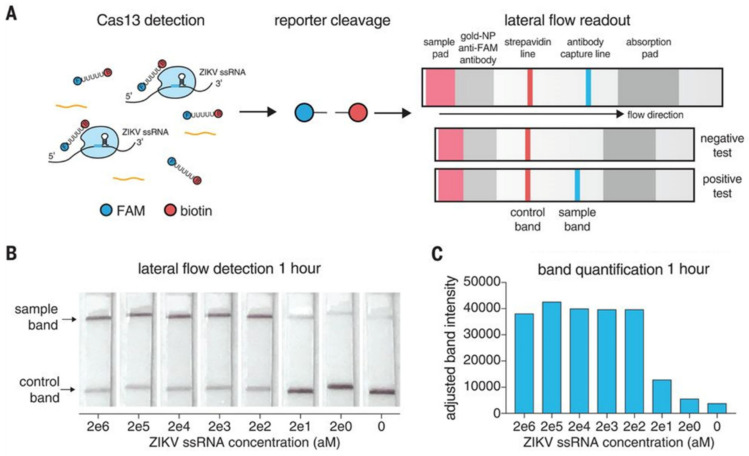
(**A**) Schematic diagram of the SHERLOCKv2 method (FAM: carboxyfluorescein, fluorophore). (**B**) Detection of synthetic Zika virus (ZIKV) RNA using the SHERLOCKv2 method by immunochromatographic strip. (**C**) Quantification of the fluorescence of the detection bands read on (**B**) linked to the presence of the FAM fluorophore, captured by its specific antibody. Adapted from [37] with permission from The American Association for the Advancement of Science, © 2018.

Introduced by Gootenberg et al., the SHERLOCK method reduced the detection limit of CRISPR–Cas13 to 10 aM (6 copies/μL), whereas the detection limit of CRISPR–Cas13 without gene amplification was 50 fM (about 30 × 10^3^ copies/μL) [3,32,33]. The detection of RNA from viruses such as the Zika virus (ZIKV), dengue virus (DENV), bacterial DNA, and cancer mutations in DNA fragments from cells have also been demonstrated using this method [3]. The method was quickly adopted by other laboratories for a variety of applications, such as the detection of Lassa and Ebola viruses [33], malaria [34], and SARS-CoV-2 [35]. A second version of the SHERLOCK platform has been developed (SHERLOCKv2) and is even more robust. It enables the simultaneous detection of 3 different RNA targets and 1 DNA target at concentrations as low as 8 zM [36]. In addition, the detection system has evolved to colorimetric reading on an immunochromatographic paper strip, which requires no special equipment (as with self-testing for SARS-CoV-2) (Figure 5). For SHERLOCKv2, amplification takes place in a single step: the biological sample is brought into contact with a solution containing crRNA–Cas13 complexes which are amplified by RPA, and then this solution is applied directly to the test strip. This was the first portable, simple, rapid, and cost-effective CRISPR–Cas-based device offering sensitivity similar to qPCR.

Many other CRISPR–Cas-based platforms have emerged, such as CARVER and CARMEN with Cas13 [29,38,39], DETECTR and HOLMES for Cas12 [40,41,42,43,44], NASBACC and CRISPREXPAR for Cas9 [19,45], and HARRY for Cas14 [46].

#### 2.2.3. Detection of Nucleic Acids by CRISPR–Cas13 Without Gene Amplification

Although the methods presented above, such as SHERLOCKv2, are highly sensitive, selective, and robust, they require gene amplification, which is not always straightforward. Furthermore, when the target to be detected is an RNA, reverse transcription into DNA is required prior to gene amplification, followed by further transcription of the amplified DNA so that it can bind to CRISPR–Cas13. All these steps can lead to a loss of measurement specificity, resulting in “false positive” results [47]. Dismissing gene amplification increases specificity and simplifies the detection method. In the case of detection without gene amplification by CRISPR–Cas, Cas13 is the most relevant class two enzyme, as it is the only one that recognizes RNA, the others recognize single- or double-strand DNA [20]. As described above, detecting RNA rather than DNA is more accurate for quantifying the evolution of an infection, whether viral or bacterial. In 2016, the first CRISPR–Cas13-based sensors without gene amplification were developed for the detection of bacteriophage RNA and human cell mRNA for concentrations down to 1 pM [32,33]. This LOD was extended to 50 fM by Gootenberg et al. for the detection of the Zika virus and dengue virus RNA [3]. Subsequently, East-Seletsky et al. succeeded in detecting RNA at a limit of 10 fM (ca. 6000 copies/μL) [48]. Qin et al. succeeded in detecting Ebola virus RNA up to 50 fM (ca. 30,000 copies/μL) [49]. All these sensors use a fluorophore-extinguisher RNA probe with a fluorescence reader to measure CRISPR–Cas13 activity. Fozouni et al. further lowered the LOD to around 1 fM (ca. 270 copies/uL) [50]. Katzmeier et al. created a pocket-sized fluorescence reader costing less than $15 that detects RNA by CRISPR–Cas13 with a LOD of 3.7 nM (number of copies unknown) [51].

Other types of CRISPR–Cas13 sensors, such as the one proposed by Johnston et al., are based on an electrochemical transduction [52]. RNA strands are linked to a redox enzyme. In the presence of CRISPR–Cas13, the RNA strands are hydrolyzed and the current measured at the electrode falls sharply because the enzyme is lifted away. This platform reaches a LOD of 4 fM (between 2000 and 7520 copies/µL depending on the targets). All these devices are detailed in Table 1.

To briefly summarize, diagnostics based on CRISPR–Cas13 technology have evolved from an experimental nucleic acid detection tool to a clinically relevant diagnostic technology for rapid, sensitive, and easy RNA detection. However, several challenges remain to transform CRISPR–Cas technology into a robust diagnostic tool for emerging diseases and pathogens. One of the main shortcomings of current CRISPR–Cas13-based diagnostics is the reliance on an RNA pre-amplification step for targets with sub-femtomolar concentrations [53]. This step adds complexity to the diagnostic, may decrease its specificity, and increases its measurement time as well as its cost [54]. In view of these issues, it is necessary to develop tools that can exploit the RNAse activity of CRISPR–Cas13 and amplify its signal. For this, field-effect transistors appear to be good candidates thanks to their capacitive coupling, which amplifies the phenomena taking place at their interfaces. These will be discussed in greater detail later in the following section.

## 3. Electrolytic Gate Field Effect Transistors (EGFETs)

### 3.1. Introduction

Electrolytic Gate Field-Effect Transistors (EGFETs) are a sub-family of FETs in which the dielectric layer is replaced by an electrolyte, which may be liquid or gel. The electrolyte used acts both as an ionic conductor and as the electrical insulator inherent in the dielectric layer of FETs. EGFETs have rapidly become important elements of advanced bioelectronics, as they are stable in aqueous media, operate at low voltages (generally between −1 V and +1 V), and can translate biological events into electrical signals with good precision [55].

**Figure 6:**
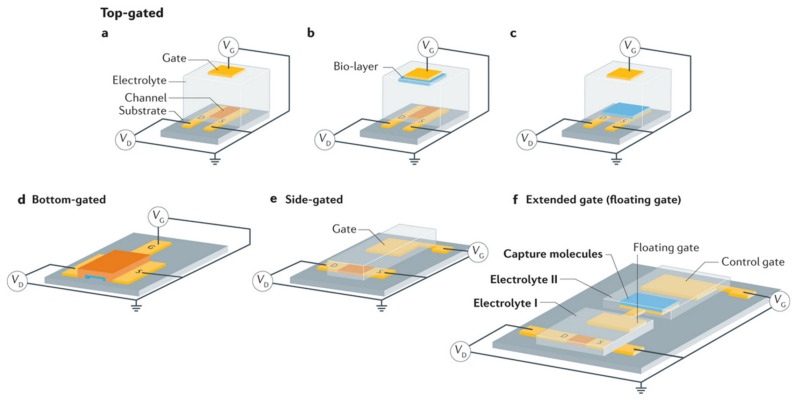
Basic architectures for electrolytic gate field effect transistors (EGFETs). The various components such as gate, electrolyte, source, drain, and semiconductor channel are illustrated. *V_G_* and *V_D_* are the respective potentials of the gate and drain, with the source connected to ground. (**a**) Top-gate configuration. (**b**) Top-gate configuration with a recognition layer on the semiconductor channel. (**c**) Top-gate configuration with a recognition layer on the gate. (**d**) Bottom-gate configuration. (**e**) Side-gate configuration. (**f**) Extended-gate (or floating-gate) configuration. Adapted from [55] from Springer Nature with permission © 2021.

Like FETs, EGFETs are three-electrode devices in which the conductivity of a semiconductor material connected between two electrodes, source and drain, is modulated by a third electrode, the gate. In a conventional EGFET structure, both the semiconductor and the gate are in direct contact with the electrolyte. Furthermore, the gate need not face the channel, as charge modulation is due to charge accumulation or depletion at the semiconductor/electrolyte interface, which is less sensitive to field line geometry as long as the ionic conductivity of the electrolyte is sufficient. In addition to the classic FET configurations where the gate is located above the semiconductor channel (top gate configuration) or below it (bottom gate configuration), it can also be coplanar (side gate configuration) or extended to an electrolyte other than that of the semiconductor channel (extended gate configuration). The latter two configurations are highly relevant as they are simpler to manufacture, functionalize, and integrate into microfluidic systems [56]. The different possible architectures for EGFETs are detailed in Figure 6.

EGFETs are classified into two distinct families, depending on the ion permeability of the semiconductor used. In the case where the semiconductor is impermeable to electrolyte ions, the application of a *V_G_* gate potential causes the displacement and accumulation of ions at the gate/electrolyte and semiconductor/electrolyte interfaces (the electrochemical double layers, EDLs). The capacitance of each interface sits between 1 and 10 µF.cm^−2^, i.e., at least ten times greater than the capacitance values obtained with conventional solid-state dielectrics [57]. This property gives rise to the main advantage of using EGFETs: their operating voltages (*V_D_* and *V_G_*) are much lower than those of OFETs (below 1 V), making them compatible with the detection of biological species in solution without the appearance of electrolysis [58].

For EGFETs using ion-permeable semiconductors, an EDL is formed only at the interface between the gate and the electrolyte, under the application of a gate potential. The ions present at the semiconductor/electrolyte interface diffuse into the volume of the semiconductor film, modulating the density of free charge carriers in the transistor channel. This process is called electrochemical doping [59]. These transistors are called organic electrochemical transistors (OECTs). When applied to the detection of DNA, all the work described to date uses a gate electrode (usually gold) functionalized with nucleic acid strands complementary to the targets to be detected [60,61,62,63].

### 3.2. Graphene-Based EGFETs (EGGFETs)

#### 3.2.1. Generalities on Graphene

Discovered by Novoselov and Geim in 2004 [64,65], graphene is a material remarkable for its optical, thermal, mechanical, and electrical properties. As the first two-dimensional material to be discovered, graphene has been the focus of much research into its fundamental properties and peculiarities. Naturally, researchers have integrated it into electronic devices [66]. Being stable in aqueous media and sensitive to field effects, it is a natural candidate for electrolytic gate transistors.

Graphene is a single-layer sheet of two-dimensional carbon atoms structured in a honeycomb lattice. The presence of non-hybridized 2p_z_ orbitals on each carbon atom enables π-π conjugation across the entire lattice, and thus, strong electron delocalization, which gives graphene its excellent electrical conductivity. Graphene is not a semiconductor but a zero-bandgap material often referred to in the literature as a semi-metal [67]. Although graphene’s bandgap is zero, the gate voltage of a transistor can still modulate the density of states in graphene: the application of a positive gate voltage induces electrons as charge carriers, while a negative voltage induces holes as charge carriers.

**Figure 7:**
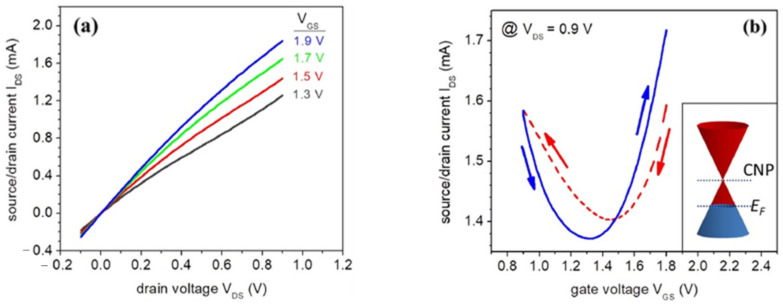
Typical (**a**) output (**b**) and transfer curves of an EGGFET with rGO as active material. Adapted from [68] with permission from Elsevier © 2021.

EGGFETs are characterized by plotting the device’s transfer and output curves. The output curves show a linear behavior representative of an ohmic operating regime (Figure 7a). The slope of the various output curves depends on the voltage applied to the gate, i.e., the charge carrier density in the rGO. The transfer curves reflect the ambipolar nature of the rGO. An increase in I_D_ is the consequence of an increase in the density of majority charge carriers, whether electrons (branch “n”, for V_G_ above a voltage denoted V*) or holes (branch “p”, for V_G_ below V*). Thus, transfer curves have the appearance of a V (or U) curve, characterized by a minimum current at potential V*. When V_G_ > V*, the current in the transistor is provided by electrons (in other words, the majority carriers are electrons), and conversely when V_G_ < V* (the majority carriers are holes). In the case of a defect-free graphene sheet, V* is ideally expected to be equal to zero, as the CNP coincides with the Fermi level. However, the Fermi level may differ from the CNP, mainly due to the presence of defects. This is the case of rGO (reduced graphene oxide) with its residual oxygen groups which contribute to p-doping and thus, to a rightward shift in the transfer curves. A typical transfer curve is shown in Figure 7b. The Dirac cone illustrates the behavior of a p-doped transistor, with a Fermi level below the CNP. Here, V* is around 1.3 V.

#### 3.2.2. Biosensing with EGGFETs

**Figure 8:**
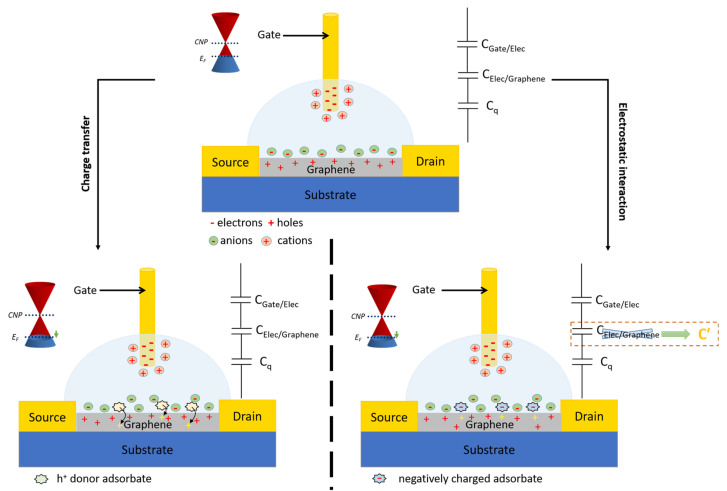
Detection mechanism of EGGFETs in the case of charge transfer (**left**) or electrostatic interaction (**right**).

For EGGFETs, the detection principle is based on the modification of the charge carrier density in the rGO either by a redox process of charge transfer or by electrostatic interactions (Figure 8). In the case of charge transfer, the target analyte (or any other redox molecule involved in molecular recognition) boosts the channel, thereby altering the Fermi level of the rGO. In the case of electrostatic interaction, the presence of a charged target analyte at the graphene/electrolyte or grid/electrolyte interface modifies the charge distribution at these interfaces, and thus, influences the charge neutrality point. As a consequence, the point V* for which I_D_ is minimal is not then located at the same potential on the transfer curve. Particular attention must be paid to this principle of electrostatic transduction, which can easily be applied to the detection of biological species, most of which are charged (proteins), or even polyelectrolyte-like (DNA, RNA). The vast majority of these sensors use transistors in which the graphene or rGO channel is functionalized by biological receptors (DNAs, antigens, antibodies).

For biosensing purposes, it is necessary to functionalize the graphene (or rGO) channel. To achieve this, it is possible to exploit the π-conjugated network, which confers π-stacking possibilities with other π-conjugated molecules. This is the case with RNA or DNA chains that possess conjugated bases and will come to bind non-covalently on graphene. Gao et al. used this property and directly functionalized a graphene channel with DNA strands, complementary to the miRNA to be detected; the sensor can detect miRNAs in 20 min with a LOD of 10 fM (6000 copies/μL) [69].

**Figure 9:**
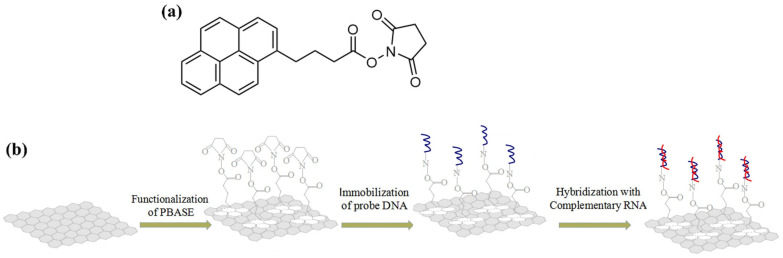
(**a**) PBASE structure and (**b**) strategy for functionalizing an EGGFET with PBASE and a DNA probe linked to PBASE by an amide function. Adapted from [71] with permission for Elsevier © 2020.

This π-stacking property can also be used to graft molecules functionalized with a π-conjugated group such as PBASE (1-pyrenebutanoic acid succinimidyl ester), which serves as the first brick for linking biological compounds. The pyrene group of the PBASE compound interacts with graphene by π-stacking, while the carboxylic acid function is used to graft biological molecules featuring a terminal -NH_2_ amine group, forming a stable amide bridge linking the two compounds. Seo et al. used this method to functionalize an EGGFET with an antibody specific to the surface protein S of COVID-19 [70]. Tian et al. also used this method to graft DNA strands and detect complementary DNA with an LOD of 0.1 fM (60 copies/μL); their approach is described in Figure 9 [71].

It has been shown that this method, and more generally the use of an adsorbent spacer group, prevents “useful” nucleic bases from interacting directly with graphene by π-stacking, and leads to improved sensitivity. For example, Chan et al. highlighted that DNA bases that interact with graphene by π-stacking are not available for hybridization with target DNA strands. Therefore, they use a larger DNA chain, comprising a section of nitrogenous bases for immobilization on graphene and a section for hybridization with the target to be detected. DNA strands hybridized without an immobilization section tend to separate from the graphene under the influence of electrolyte flow, which is not the case for DNA strands immobilized by an ad hoc immobilization segment [72].

Graphene can also be functionalized covalently. “Formic acid” graphene and “acetic acid” graphene are two compounds of interest for bioreceptor grafting [73,74]. Both of these compounds have carboxylic acid groups that can be used to covalently link biological targets with an accessible -NH_2_ amine function. Hensel et al. demonstrated the grafting of aptamers onto “acetic acid” graphene [73]. The authors reported that due to these functions, the distance between graphene sheets is larger, which avoids problems of steric hindrance and accessibility during amide bridge formation.

**Figure 10:**
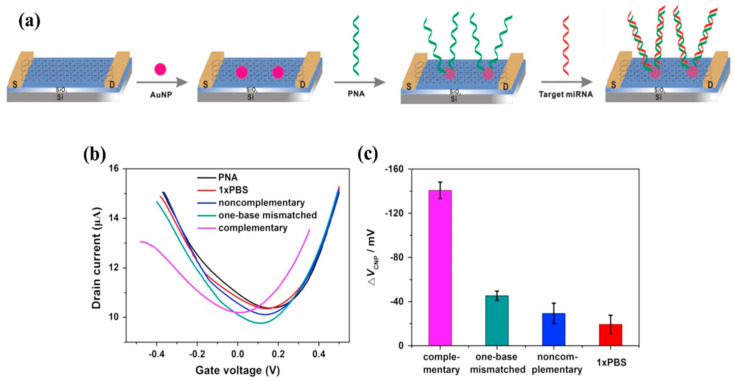
(**a**) Simplified process for functionalizing the channel of an EGGFET for DNA detection described by Cai et al. (**b**) Transfer curves (the PNA curve corresponds to the functionalized transistor) and (**c**) shifts in the associated ΔV_CNP_ charge neutrality point as a function of the different species brought into contact with the transistor: PBS buffer without target strand (red), PBS + non-complementary DNA (navy blue), PBS + DNA carrying a mismatch (light blue), and PBS + complementary DNA (purple). Adapted from [76] with permission from Elsevier © 2015.

Another approach to the covalent functionalization of graphene involves the use of gold nanoparticles (AuNPs), which are spontaneously reduced on graphene (which is a reducing agent, its oxidized form being graphene oxide, GO). AuNPs are generally formed by contacting graphene (or rGO) with an aqueous solution of chloroauric salts such as HAuCl_4_, where gold is in Au(III) oxidation state. On contact with graphene, Au(III) is spontaneously reduced to Au(0) by a sp^2^ carbon atom, given that the redox potential of Au(III) is 1.0 V and that of graphene 0.22 V (vs. ESH) [75]. AuNPs serve as anchoring points for biological molecules with a thiol group -SH. The main advantage of this functionalization route over those based on π-stacking interactions is that it spaces the probe from the graphene, making it more available. This approach was used by Cai et al. to detect RNA by hybridization with a complementary PNA (peptide nucleic acid, uncharged) strand with an LOD of 10 fM (6000 copies/μL) [76], and is described in Figure 10.

**Figure 11:**
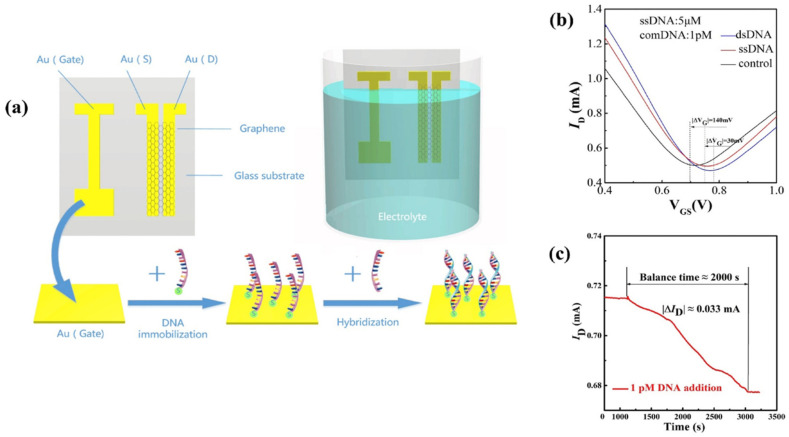
Operating principle of the DNA-sensing EGGFET described by Li et al. (**a**) Structure and functionalization of the EGGFET. (**b**) Transistor transfer curves measured with a bare gate (control), after functionalization with single-stranded DNA (ssDNA), and after hybridization with its complement (dsDNA). (**c**) Evolution of the current response of the functionalized EGGFET during hybridization with the complementary DNA chains. Gate and drain potentials are fixed at V_G_ = 0.8 V and V_D_ = 0.1 V. Adapted from [77] with permission from © 2019 Elsevier B.V. All rights reserved.

Rather than functionalizing the graphene (or rGO), it is also possible to functionalize the gate electrode. However, the gate of EGGFET transistors is not always functionalizable, as it is often replaced by a non-polarizable Ag/AgCl reference electrode, which makes it possible to control the actual potential applied whatever the electrolyte solution contains, and thus, to make measurements more reproducible. Nevertheless, some works such as those by Li et al. describe the use of a gold grid functionalized with DNA strands (Figure 11) [77]. Charged species recognition phenomena at the grid/electrolyte interface modify the charge arrangement and influence the electric field, inducing an evolution of the transistor output current I_D_. A LOD of 1 fM (600 copies/μL) has been obtained for DNA detection by these authors [77].

To conclude this section on EGFETs, graphene-based EGFETs are the most popular for stability reasons, and also because graphene is easier to functionalize than other active materials such as inorganic or organic semiconductors. Stability is crucial in the field of DNA detection because processing and reaction times are quite long, so the materials that make up the transistors can degrade, and their instability affects the reliability of the measurements. In addition, the 2D nature of graphene, i.e., the highest surface-to-volume ratio expected, brings a particular sensitivity to its chemical environment, be it electrostatic charges carried by neighboring molecules or the presence of donating or withdrawing groups capable of transferring charges to/from the graphene layer. In other words, EGGFETs are particularly well suited to transducing events involving DNA or RNA, highly charged molecules (phosphate backbone) with known charge transfer capabilities (nucleobases). As shown, the signal that can be extracted from DNA EGGFETs is based on the shift (in potential) of their transfer curves, ultimately amplified into a current (by the design of any transistor). To further improve sensitivity, a further amplification stage based on enzymatic activity can be added. The most common approach is to couple FETs (generally OECTs in this case) to DNA labeled with an enzyme (e.g., glucose oxidase from horse radish peroxidase). Recognition of a labeled target by a fixed probe brings the enzyme close to the transistor, generating a redox current that is amplified. Another more recent and original approach is to use CRISPR/Cas activity. First, it is known to improve hybridization efficiency and specificity compared to simple DNA/DNA or DNA/RNA hybridization. Second, because it has enzymatic cleavage activity, it brings molecular amplification, as a single hybridization event generates a cascade of chemical events. These approaches are described in detail in the following section.

## 4. CRISPR–Cas and Transistors

CRISPR–Cas complexes are relevant systems for biosensing because they involve two sites for recognition and hydrolysis of nucleic acid strands, as detailed in Section 2.2. The first property, called cis-cleavage, is the one mainly used by Cas9, and consists of the recognition of a target DNA by a guide RNA previously bound to the enzyme. The target DNA strand (or RNA) then hybridizes to the guide RNA; this step may be followed by hydrolysis of the target DNA chain, as with Cas9. However, hydrolysis does not always take place, and target DNA/guide RNA hybridization can also serve as a trigger for the appearance of another catalytic site, as in Cas12, Cas13, and Cas14. Once activated by hybridization between target DNA and guide RNA, these enzymes undergo structural reformation to reveal a new catalytic site, triggering non-specific RNAse (or DNAse) activity (i.e., any strand of RNA or DNA in the vicinity of the enzyme is hydrolyzed, without specificity for any particular sequence). This property is known as trans-cleavage. These two properties of Cas enzymes can be exploited in EGFET transistors, based on specific recognition of a target RNA (cleavage-cis) and amplification of the signal by the RNAse activity of these enzymes (cleavage-trans). The various sensors that use the combination of CRISPR–Cas complexes and EGFETs to detect biological species are discussed below.

### 4.1. Exploiting Cis-Cleavage

**Figure 12:**
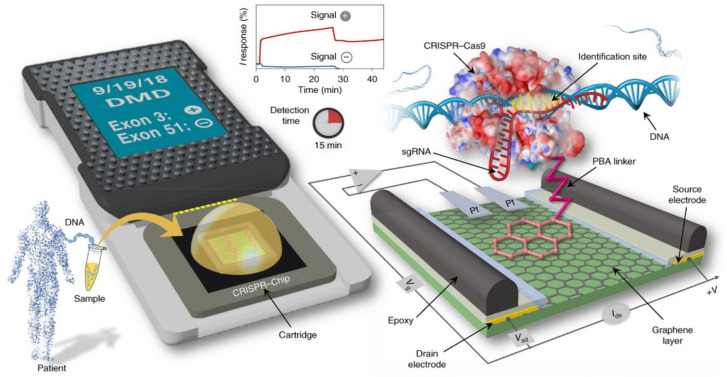
CRISPR-Chip diagnostic platform proposed by Hajian et al. enables DNA detection in less than 15 min. The dCas9 enzyme complexed with a DNA target-specific guide RNA is immobilized on the graphene surface of an EGGFET. The immobilized CRISPR–dCas9 complex scans the entire genomic DNA until it identifies its target sequence. Hybridization between the target DNA and CRISPR-dCas9 modulates the electrical characteristics of the EGGFET, including the I_D_ output current. Target DNA detection is possible with a LOD of 1.7 fM (ca. 1000 copies/μL). Reproduced from [78] with permission from Springer Nature © 2019.

Cas9 has no trans-cleavage activity, but only a cis-cleavage property through recognition of double-stranded DNA at its guide RNA. This property of recognizing a specific sequence in a double-stranded DNA genome is at the heart of CRISPR–Cas technology. The first transistor using Cas9 and EGFETs was described in 2019 by Hajian et al. (Figure 12) [78]. The Cas9 enzyme used is first deactivated, to prevent target DNAs from hydrolysis after guide RNA-target DNA hybridization. This deactivated Cas9 enzyme is referred to as dCas9. CRISPR–dCas9 complexes are immobilized on the channel of an EGGFET via a PBA (pyrene butanoic acid) group. An amide bridge is created between the acid function of the PBA and a free -NH_2_ end of CRISPR-dCas9 to bind the enzyme to the EGGFET. The sensor’s detection principle is based on hybridization between the target DNA and CRISPR-dCas9 immobilized on the channel surface.

In the context of a conventional transistor, biorecognition events taking place at a distance greater than the Debye length λ (λ ≈ 1 nm under salinity conditions equivalent to phosphate-buffered saline, PBS) are not efficiently translated because the charges carried by the targets and probes are shielded by free ions in solution. Since Cas enzymes are large riboprotein complexes (>10 nm), hybridization of the DNA target to the guide RNA therefore takes place outside the Debye length λ; yet it is quantifiable by an evolution of the output current of the I_D_ transistor thanks to the Donnan effect [79].

Indeed, the CRISPR-dCas9 protein layer immobilized on the EGGFET surface can be considered an ion-permeable and selective membrane. Hybridization of the negatively charged DNA target creates an ion-dense layer on the membrane surface, due to the accumulation of cations to maintain charge neutrality. This change in ion concentration between the electrolyte volume and the membrane creates a Donnan potential. This new potential modifies the electric field between the grid and the channel, which in turn modulates the output current. This phenomenon makes it possible to detect biological events at distances greater than the Debye length [80]. It is this effect that also enables the detection of antigen–antibody interactions on the surface of a transistor. The CRISPR–Chip sensor by Haijian et al. sense target DNA in 15 min with a LOD of 1.7 fM (ca. 1000 copies/μL).

**Figure 13:**
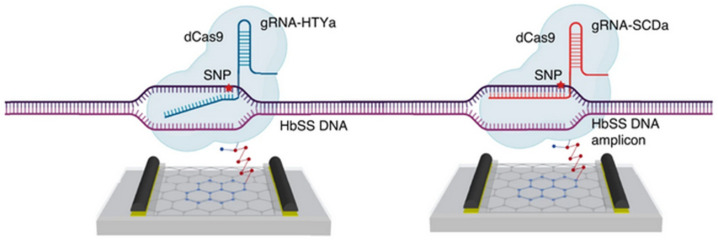
Schematic diagram of the CRISPR-SNP-Chip transistor functionalized with dCas9, proposed by Balderston et al. gRNA-HTYa and gRNA-SCDa are the guide RNAs. In the presence of the single polymorphism associated with the gRNA-SCDa guide RNA, dCas9-HTYa does not hybridize completely with its target DNA, which then dissociates from the dCas9–gRNA complex. Adapted from [81] with permission from Springer Nature © 2021.

This sensor was improved by Balderston et al. into a device called CRISPR-SNP-Chip, capable of discriminating against the mutation of a single base pair in a target DNA [81] (Figure 13). This is made possible by the excellent selectivity brought by the CRISPR/Cas system compared to conventional (naked) DNA/DNA hybridization. In addition to the output current I, two other electrical parameters are analyzed: (1) The capacitance C of the graphene/electrolyte interface which changes following hybridization of the target DNA to the CRISPR–dCas9 and influences the slope of the transfer curve at a given potential; (2) The position of the minimum V* of the transfer curve. By exploiting the relative variations in these three parameters (I, C, and V*), these authors created a very sensitive sensor that can discriminate a one-base mismatch within 1 h, exemplified for the genomic DNA of Amyotrophic Lateral Sclerosis (ALS) and sickle cell disease [81].

**Figure 14:**
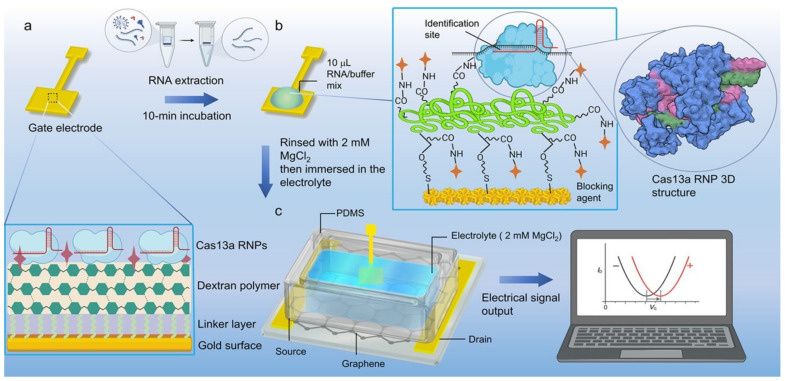
Design of the CRISPR–Cas13a-based sensor proposed by Yu et al. (**a**) Functionalization of the transistor gate by CRISPR–Cas13a. (**b**) Detection principle between RNA target and CRISPR–Cas13a. (**c**) Sensor structure with interchangeable gate. Characterization of the biological signal into an electrical signal by the EGGFET transistor. Reproduced with permission from [82]. Copyright © 2022, American Chemical Society.

Two other works by Li’s group reported the use of cis-cleavage of CRISPR–Cas complexes for biosensing [82,83]. In contrast to the previous example, the CRISPR/Cas system is grafted on the gate of the transistor. Two sensors are proposed, one based on Cas13 and the other based on Cas12, both grafted onto the gate via a self-assembled layer and/or a polymer layer (the functionalized gate structures are detailed in Figure 14). The pairing of negatively charged target DNA (or RNA) on CRISPR–Cas12a (or CRISPR–Cas13a) modifies the arrangement of charges at the gate/electrolyte interface, inducing an evolution of the electric field, which induces a shift in the transfer curve towards positive potentials.

Their Cas12a-based sensor is capable of detecting Papillomavirus DNA in 20 min with an LOD of 8.3 aM (5 copies/μL) [83] while the Cas13-based one detects SARS-CoV-2 RNA in 10 min with an LOD of 13 aM (8 copies/μL) [82]. The main advantage of the functionalized gate is two-fold: it gives the possibility to change it according to the target to be detected, independently of the graphene side. It is also easier to modify the surface of a metal electrode than to functionalize graphene (or a semiconductor). However, using the gate as a sensitive electrode can also be a disadvantage. Indeed, it precludes the use of a pseudo-reference electrode (such as an Ag/AgCl) as a gate, which is known to significantly stabilize the potentials and make such transistors more stable.

Some RNA or DNA sensors based on the cis-cleavage property of Cas enzymes immobilized on one of the two interfaces of an EGGFET are listed in Table 2.

### 4.2. Exploiting Trans-Cleavage

The trans-cleavage property is the most interesting for the creation of point-of-care (POC) sensors, as it enables molecular signal amplification. Indeed, a single RNA target activates a CRISPR–Cas13 enzyme capable of hydrolyzing a large amount of RNA strands and not only one [24]. This phenomenon of enzymatic amplification of the signal is very interesting for biosensing, compared with previous generations of sensors based on the hybridization of a single RNA probe with a single target, for limited molecular reorganization and therefore a limited signal. The advantage of CRISPR–Cas13 is that the output signal is multiplied by the hydrolysis activity, since many strands of RNA are hydrolyzed, for a much greater (macro)molecular reorganization. It is precisely this trans-cleavage property that enhances the sensitivity of the devices presented above, while specificity is ensured by cis-cleavage of the target RNAs on the CRISPR–Cas enzymes to which they are specific. Clearly, as Cas9 does not exhibit trans-cleavage activity, its use is not possible for this purpose.

Before being used in transistors, CRISPR–Cas13 trans-cleavage was used for electrochemical sensors. This was introduced by the work of Dai et al. in 2019, who proposed an electrochemical sensor named E-CRISPR (Electrochemical CRISPR) for the detection of human papillomavirus and parvovirus B-19, with lower (picomolar) sensitivity [84]. Single-stranded DNAs are grafted onto the working electrode and carry a redox probe at its free end. Hydrolysis of these DNA strands by CRISPR–Cas12a releases the redox probes into the electrolyte and reduces the redox current of the probe at the electrode surface. Zhang et al. used the same detection principle with a hairpin RNA probe to bring the redox probe closer to the electrode surface [85]. This sensor is also used for human papillomavirus detection, with a dynamic detection range between 50 pM and 100 fM (60,000 copies/μL). Recently, other electrochemical sensors capable of detecting RNA concentrations with an LOD of 10 aM (6 copies/μL) have emerged [86,87]. This is notably the case of the sensor proposed by Lee’s group, which is also based on RNA strands bound to an electrode, at the end of which a redox probe is grafted [88]. The sensor’s response time is relatively long, around 3 h, which gives CRISPR–Cas13 enough time to hydrolyze the RNA strands and obtain an LOD at the attomolar level.

In the following sub-sections, the use of Cas12 and Cas13 in EGGFETs are discussed.

#### 4.2.1. DNA Detection by Cas12

As with those presented above, most devices combining CRISPR–Cas12 and EGFETs use graphene-based transistors. In the case of DNA detection, Cas12, and Cas14 enzymes can be used but no paper has yet described the use of Cas14 with EGGFETs. With regard to articles employing CRISPR–Cas12, the channel is an interface of choice for functionalization (as explained above, because it allows the use of an Ag/AgCl gate [89], which makes the transistors more stable with time.

**Figure 15:**
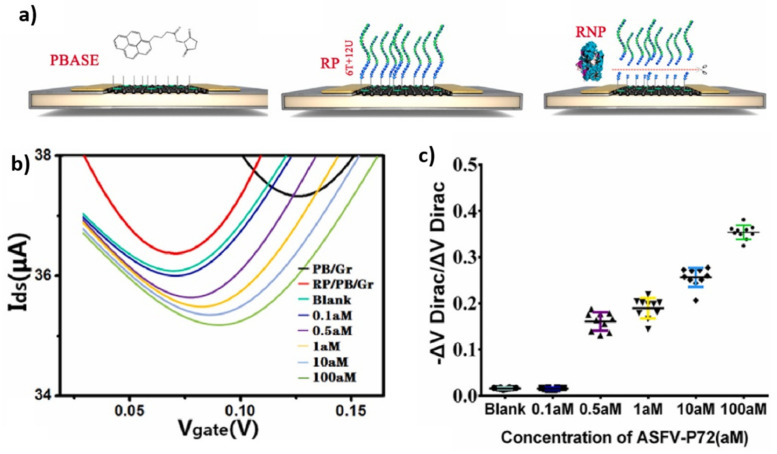
Design of the CRISPR–Cas13a-based sensor proposed by Yu et al. (**a**) Functionalization of the transistor gate by CRISPR–Cas13a. (**b**) Detection principle between RNA target and CRISPR–Cas13a. (**c**) Sensor structure with interchangeable gate. Characterization of the biological signal into an electrical signal by the EGGFET transistor. Reproduced with permission from [92], © 2023 Elsevier.

Within the framework of EGGFETs that exploit the enzymatic activity of Cas12, all employ a non-covalent grafting route through the use of PBASE (with the great advantage of preserving the electrical properties of graphene), detailed above. Although the mode of channel functionalization remains the same for these different works, the DNA probes immobilized on the transistor channel are different. Wang et al. use a 20-nucleotide T DNA probe for the detection of monkeypox (MPXV). Their sensor has an LOD of 1 aM and can differentiate between the different mutations of this disease in 20 min at an operating temperature of 52 °C [90]. Weng et al. used 20-nucleotides poly-A or poly-C DNA probes to detect human papillomavirus (HPV-16) and the Escherichia coli membrane gene with LODs of 1 aM and 10 aM (6 copies/μL), respectively [91]. This detection is possible in less than 30 min, and the incubation of CRISPR–Cas12a on the transistor is performed at room temperature. Their sensor can also differentiate a single mismatch on Escherichia coli plasmid DNA and allows an LOD of 1 pM. For their part, Wang et al. chose to modify the transistor-grafted probe to increase the sensitivity of their device [92]. They used an RNA consisting of 6 T nucleotides followed by 12 U nucleotides (detailed in Figure 15) which involves the hydrolysis by CRISPR–Cas12 of only the T part of this DNA–RNA hybrid probe (CRISPR–Cas12 does not hydrolyze polyU sequences). By designing the RNA so that this 6T part is close to the channel, this strategy increases the sensitivity of the device compared with a situation where the cleavage occurs randomly along the RNA strands. The LOD obtained, for African swine fever virus (AFSV), is 0.5 aM (1 copy/3 μL), with a 30 min incubation at room temperature. This is the lowest detection limit described to date for DNA detection using EGGFETs.

#### 4.2.2. RNA Detection by Cas13

**Figure 16:**
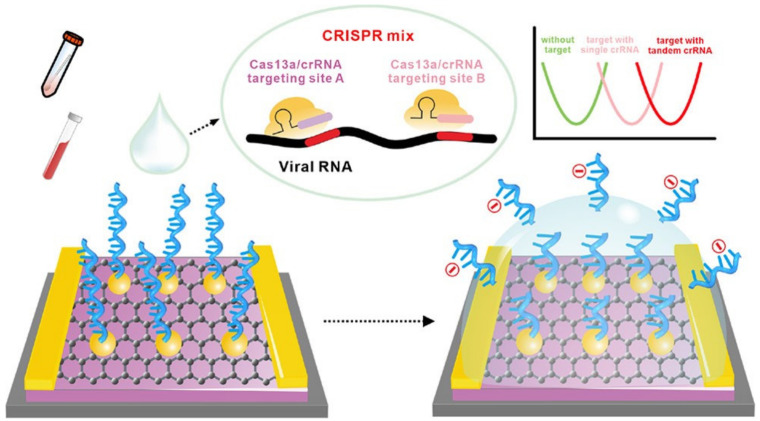
Schematics of the biosensor proposed by Li et al. which uses two different crRNAs for accelerated and more sensitive detection of SARS-CoV-2. The RNA probes present a hairpin structure. Reproduced with permission from [93]. Copyright © 2022, American Chemical Society.

Numerous sensors featuring EGGFETs and CRISPR–Cas13 have been developed in the wake of the COVID-19 pandemic. The first device was developed by Li et al. in 2022 for the detection of the ORF1ab and N genes of SARS-CoV-2 (but was also exemplified with hepatitis C virus, HCV) [93]. The transistor used is an rGO-EGFET, where the rGO channel is functionalized by gold nanoparticles onto which RNA probes are grafted (Figure 16). The chosen RNA probes have a hairpin structure and are oriented horizontally rather than vertically, maximizing the density of negative charges closest to the rGO, within the Debye length. This sensor is capable of detecting RNA after a 20 min incubation at 37 °C and achieves an LOD of 1.6 aM (1 copy/μL) for SARS-CoV-2.

**Figure 17:**
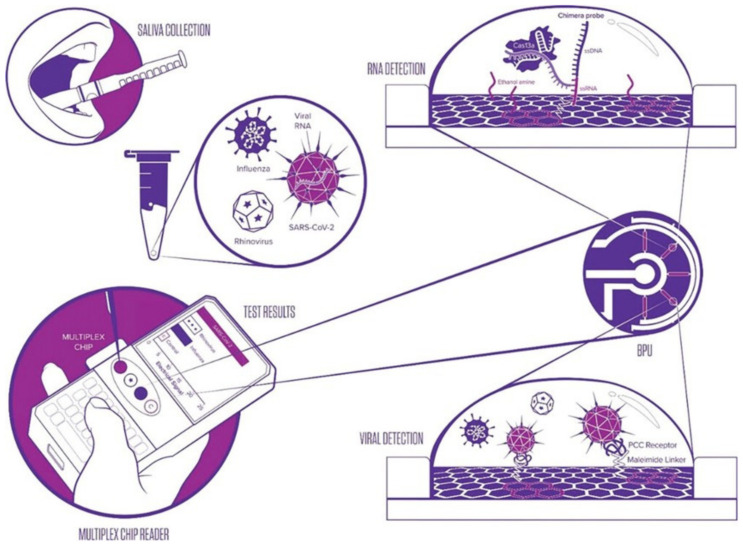
Schematics of the EGGFET proposed by Ban et al. for the detection of SARS-CoV-2 RNA and virus particles. Reproduced with permission from [95], © 2023 John Wiley and Sons.

Other EGGFET-based sensors for the detection of SARS-CoV-2 have been developed, such as those proposed by Li [94], Ban [95], and Sun [96]. These three sensors use an EGGFET on which RNA probes are embedded using PBASE (i.e., immobilized onto the graphene active layer). The sensor proposed by Ban et al. targets SARS-CoV-2 RNA with an LOD of 65 aM (40 copies/μL) using CRISPR–Cas13a (Figure 17), whereas the system developed by Li et al. has been validated for the detection of SARS-CoV-2 and respiratory syncytial virus (RSV), with an LOD of 1 aM (6 copies/10 μL) after an incubation time of 30 min at 37 °C. The RNA probes used on these two sensors are 20-nucleotide chains (polyU20). Like the polyU-polyT DNA–RNA hybrid probes proposed by Hu et al. for DNA detection using Cas12, Sun et al. have also proposed an RNA–DNA hybrid probe for RNA detection using Cas13 [96]. This probe is composed of 6 U nucleotides followed by 12 T nucleotides, which directs Cas13 activity towards the U part of this probe and accelerates signal detection. An incubation time of 2 h at 37 °C and an LOD of 0.25 aM (3 copies/20 μL) were obtained.

In a view of comparison, Chen et al. have described the use of an EGFET based on the inorganic semiconductor IGZO (oxide of zinc, gallium, and indium), in a device named CAVRED (CRISPR-based Amplification-free Viral RNA Electrical Detection) [97]. The IGZO layer is functionalized with APTES ((3-aminopropyl)triethoxysilane) to create amine functions that can bind to RNA probes. In a similar way to EGGFETs, hydrolysis of RNA probes by Cas13 generates a change in charge density at the channel surface that modifies the I_D_ drain current. The CAVRED platform has been validated for the detection of SARS-CoV-2 with an LOD of 1 copy per µL (≈1.7 aM).

The various EGGFET-based biosensors exploiting the trans-cleavage property of the CRISPR–Cas systems presented above are grouped together in Table 3 with their different characteristics.

Quite intuitively (and comparing Table 3 with Table 2), it appears that devices using trans-cleavage of CRISPR–Cas complexes are more sensitive than those based on cis-cleavage interaction alone because not only the CRISPR–Cas 13 allows a faster hybridization of the probes onto the target strands (what CRISPR–Cas12 does) but its enzymatic activity brings an additional molecular amplification upon recognition, which cumulates with the intrinsic electrical amplification behavior of the transistor.

## 5. Conclusions

The COVID-19 pandemic highlighted the lack of rapid, real-time, and decentralized nucleic acid (DNA or RNA) detection methods. Today, PCR is undoubtedly the gold standard. However, some CRISPR–Cas ribonucleic complexes, discovered in 2007, have recently been able to compete with PCR in terms of detection limits thanks to their trans-cleavage activity, which can amplify the output hybridization signal by several orders of magnitude. This has revolutionized nucleic acid detection and some devices, such as SHERLOCK, have been rapidly cleared by the FDA for SARS-CoV-2 detection. However, these detection platforms still require a pre-amplification step that is difficult to compare with the design of POC devices. The improvements made in recent years demonstrate that CRISPR–Cas technology can be combined with the most advanced and reliable electrolyte-gated transistors, such as the graphene-based ones (EGGFETs), making this pair a plausible candidate for the creation of POC devices for rapid, sensitive, and specific detection. As explained and shown in this review, on the one hand, CRISPR–Cas offers the advantage of an enzymatic activity that locally generates molecular amplification in situ. On the other hand, transistors are devices that are able to electrically amplify a weak signal (e.g., a local change is potentially induced by the arrival or departure of a charged species such as DNA, a change in the conformation of DNA strands, or the cleavage of DNA or RNA strands) into a stronger electrical signal (whether voltage or current). A cumulative effect is achieved by bringing the CRISPR/Cas particles as close as possible to the sensitive part of the transistor, i.e., the gate or channel. It should be remembered that immobilization carried out on the gate or the channel has its respective advantages and disadvantages: gate functionalization is chemically simple, but it forbids the use of a pseudo-reference as a gate, while channel functionalization allows to take better advantage of the sensitivity of 2D materials such as graphene but exposes the transistor to potential instability. As far as the functionalization strategy is concerned, recent works have adopted the strategies previously developed for electrochemical (redox) DNA sensors: immobilization of DNA with secondary structures such as hairpins or more complex superstructures, CRISPR–Cas hydrolysis directed close to the surface rather than far away by insertion of U or T sequences. CRISPR–Cas in the context of transistors is now a very dynamic field that needs to be followed closely.

## Figures and Tables

**Table 1:** Overview of different sensors using CRISPR–Cas13 for RNA detection without gene amplification.

Name of the Method	Pathogen	Preamplification	Measurement	LOD	Refs
SHERLOCK	ZIKV, DENV	RT-RPA	Fluorescence	10 aM (6 copies/μL)	[3]
SHERLOCKv2	ZIKV, DENV	RT-RPA	Fluorescence	8 zM (unknown copies/μL)	[37]
			Colorimetric strip	2 aM (1–2 copies/μL)	[37]
-	Bacteriophage RNA, Human RNA	None	Fluorescence	1 pM (1500 copies/μL)	[32,33]
-	ZIKV, DENV	None	Fluorescence	20 aM (12.5 × 10^3^ copies/μL)	[3]
-	Ebola	None	Fluorescence	50 fM (6 × 10^4^ copies/μL)	[49]
-	Not specified	None	Fluorescence	10 fM (unknown copies/μL)	[44]
-	SARS-CoV-2	Naone	Fluorescence on smartphone	1 fM (270 copies/μL)	[50]
-	SARS-Cov-2 (Gene E)	None	Electrochemical	4 fM (2000 and 7520 copies/µL)	[52]

**Table 2:** Summary of the various sensors proposed for nucleic acid detection, which exploit the cis-cleavage property of Cas enzymes. All the transistors used in these devices are EGGFETs.

Enzyme	Target	Functionalization Area	Grafting Scheme	LOD	Reaction Time	Refs
dCas9	ADN	Channel	Graphene/PBA/Cas	1.7 fM (1000 copies/μL)	15 min	[78,81]
Cas12a	ADN	Gate	Au/SAM/Cas	8.3 aM (5 copies/μL)	20 min	[83]
Cas13a	ARN	Gate	Au/SAM/Dextran/Cas	13 aM (8 copies/μL)	10 min	[82]

**Table 3:** An overview of the various EGGFETs proposed for nucleic acid detection using the trans-cleavage property of Cas enzymes.

Enzyme	Target	Transistor	Temperature	LOD	Reaction Time	Refs
Cas12b	MPXV	Graphene-EGFET	52 °C	1 aM(6 copies/10 μL)	20 min	[90]
Cas12a	AFSV	Graphene-EGFET	TA	0.5 aM(6 copies/20 μL)	30 min	[92]
Cas12a	HPV-16	Graphene-EGFET	TA	1 aM(6 copies/10 μL)	30 min	[91]
Cas13a	SARS-CoV-2 HCV	rGO-EGFET	37 °C	1.56 aM(1 copy/μL)	30 min	[93]
Cas13a	SARS-CoV-2	Graphene-EGFET	37 °C	0.25 aM(3 copies/20 μL)	120 min	[96]
Cas13a	SARS-CoV-2 RSV	Graphene-EGFET	37 °C	1 aM(6 copies/10 μL)	30 min	[89]
Cas13a	SARS-CoV-2	Graphene-EGFET	TA	65 aM	30 min	[95]
Cas13a	SARS-CoV-2	IGZO-EGFET	TA	1.7 aM(1 copy/ μL)	20 min	[97]
